# Dental Education

**Published:** 1873-11

**Authors:** 


### EDITORIAL, ETC.

*Dental Education.*—The dental colleges throughout the country are now in full operation, so far as the preliminary course is concerned, the regular lectures in the majority of them beginning on the 1st of November.

Owing to the financial crisis the attendance at both medical and dental colleges may be somewhat affected during the present session ; but so far, the session at the Baltimore College of Dental Surgery has opened with a very good prospect for a large class, the number of students at the time we write (October 27th,) being in excess of some previous years, and nearly equal to last year, when there was a large class in attendance. Taking into consideration the financial condition of the country, and especially of the Southern States, the friends of this institution are very much gratified at this evidence of its prosperity, especially as the majority of the second course students enter on or near November 1st of each year. Among the number already matriculated are four students from foreign countries.

Dr. Taft, in the October No. of *Dental Register*, truly remarks that " the subject of dental education is one that ought to elicit the highest interest of every dentist. Every one should feel the importance of the subject enough to examine the character and standing of our educational institutions, and seek, where there are defects, to have them corrected, and sustain those that are most efficient in the work.

" Unfortunately there is a great want of interest in this matter, and in many cases not only a lack of interest, but an active opposition, more or less pronounced, either in word or act. Many dentists will discourage students, and often prevent them from entering a dental college ; affirming that they will have better opportunities for obtaining the desired knowledge in an office. Others manifest an entire indifference as to whether their students take a college course or not, giving them little or no preparation for it, and manifesting little or no interest what institution, if any, the student enters.

"There are other influences constantly operating to prejudice the best interests of professional education, such as an over-anxiety to secure large classes, and to graduate the largest number, irrespective of the character of the material composing the classes.

"An emulation as to who shall accomplish the greatest good is always laudable; a purpose is always best accomplished by doing thoroughly that which is attempted."

